# Anemia and thrombocytopenia in people living with HIV/AIDS: a narrative literature review

**DOI:** 10.1093/inthealth/ihaa036

**Published:** 2020-07-04

**Authors:** Amanda Marchionatti, Mariana Migliorini Parisi

**Affiliations:** Centre for Health and Rural Sciences, University of Cruz Alta, RS, Brazil; Centre for Health and Rural Sciences, University of Cruz Alta, RS, Brazil

**Keywords:** AIDS, anemia, erythrocytes, HIV, platelets, thrombocytopenia

## Abstract

Hematologic changes are frequent complications in people living with HIV/AIDS (PLWHA). Anemia and thrombocytopenia are the most frequent multifactorial hematologic abnormalities and are associated with a low quality of life and high death rates. This study aims to describe the prevalence of anemia and thrombocytopenia in PLWHA and to identify the main clinical characteristics that aggravate these conditions in studies published in the last 10 y. A comprehensive search was performed on the PUBMED database, using the terms ‘HIV infection *and* anemia’ and ‘HIV infection *and* thrombocytopenia’. Additional searches were made in the reference lists of articles covering the theme. The selected studies reported an overall prevalence of anemia from 7.2% to 84% and of thrombocytopenia from 4.5% to 26.2%. The prevalence of thrombocytopenia and anemia were aggravated by a CD4^+^ T lymphocyte count of <200 cells/μL, increased viral load and coinfections or opportunistic infections. Antiviral therapy (ART) shows a beneficial effect, reducing the frequencies of thrombocytopenia and anemia, except in a zidovudine-based ART regimen, which worsens the anemic condition. Because anemia and thrombocytopenia are treatable comorbidities associated with increased mortality among PLWHA, physicians should monitor these risk factors in order to establish better interventions and reduce morbidity and mortality in PLWHA.

## Introduction

HIV is a retrovirus that causes a multi-systemic disease because of viral action on cells that express CD4 protein in their cytoplasmic membrane. The progressive immunodeficiency caused by the decrease of CD4^+^ T lymphocytes during HIV infection is responsible for the installation of AIDS, leading the individual to a predisposition to opportunistic infections and a lower quality of life.^1^ Approximately 38 million individuals suffer from HIV worldwide, with 70% of those infected living in sub-Saharan Africa. AIDS is one of the main causes of premature death in the world.^2^

Hematological changes are one of the most common complications among people living with HIV/AIDS (PLWHA).^3^ The bone marrow, the site of blood formation, is the target of the combined effect of the HIV viral infection, the drugs used during the AIDS treatment, the inflammatory mediators released during infection and the effects of likely opportunistic pathogens. The direct and indirect effects of HIV infection on hematopoietic progenitor cells impair bone marrow homeostasis, affecting cell proliferation and differentiation during hematopoiesis. The main consequences are altered cellularity with reduction of all hematological lineages, dysplastic changes in the erythroid and granulocytic series, megaloblastic abnormalities in the erythroid series and reticulum endothelial iron block. In addition, hematopoietic progenitor cells express CD4 receptors, type 4 C-X-C chemokine receptors and type 5 chemokine receptors, making them susceptible to being infected by HIV.^4^^,^^5^

The effects of HIV on bone marrow mainly cause anemia and thrombocytopenia in peripheral blood, clinical conditions related to the increased risk of death in PLWHA.^6^ Therefore, it is essential to monitor the evolution of the blood count during HIV infection, in order to detect the development of these hematological disorders and perform the necessary clinical interventions to avoid comorbidities.^7^

In recent years, studies conducted in different locations around the world have shown highly different results regarding changes in erythrocytes and platelets in PLWHA.^3^^,^^8^^-^^10^ In addition, there are inconsistencies about the way in which the patient's clinical characteristics may aggravate or attenuate hematological manifestations, causing great heterogeneity between studies.^6^^,^^7^ The introduction of antiretroviral therapy (ART) also seems to have a dichotomous effect on the hematological compartment, which, in some cases, may in fact exacerbate anemia.^11^ Given the diversity of results, it becomes essential for the data available in the literature to be reviewed to demonstrate the complex effects of HIV on the hematopoietic system.

In this study, we established two questions in order to raise qualitative data that enable a comprehensive assessment of the problem of planning interventions that mitigate the effects of hematological changes in the progression of AIDS: (1) What is the prevalence of anemia in PLWHA in different locations worldwide and in different clinical conditions? and (2) What is the prevalence of thrombocytopenia in PLWHA in different locations worldwide and in different clinical conditions? Therefore, the objective of this study was to describe the prevalence of anemia and thrombocytopenia in PLWHA and to identify the main clinical characteristics that aggravate these conditions in studies published in the last 10 y.

## Methods

A narrative review of literature was performed on the PUBMED database from January to March 2020. The searches used the terms ‘HIV *and* anemia’ and ‘HIV *and* thrombocytopenia’ and the filter ‘10 year’.

**Figure 1.**
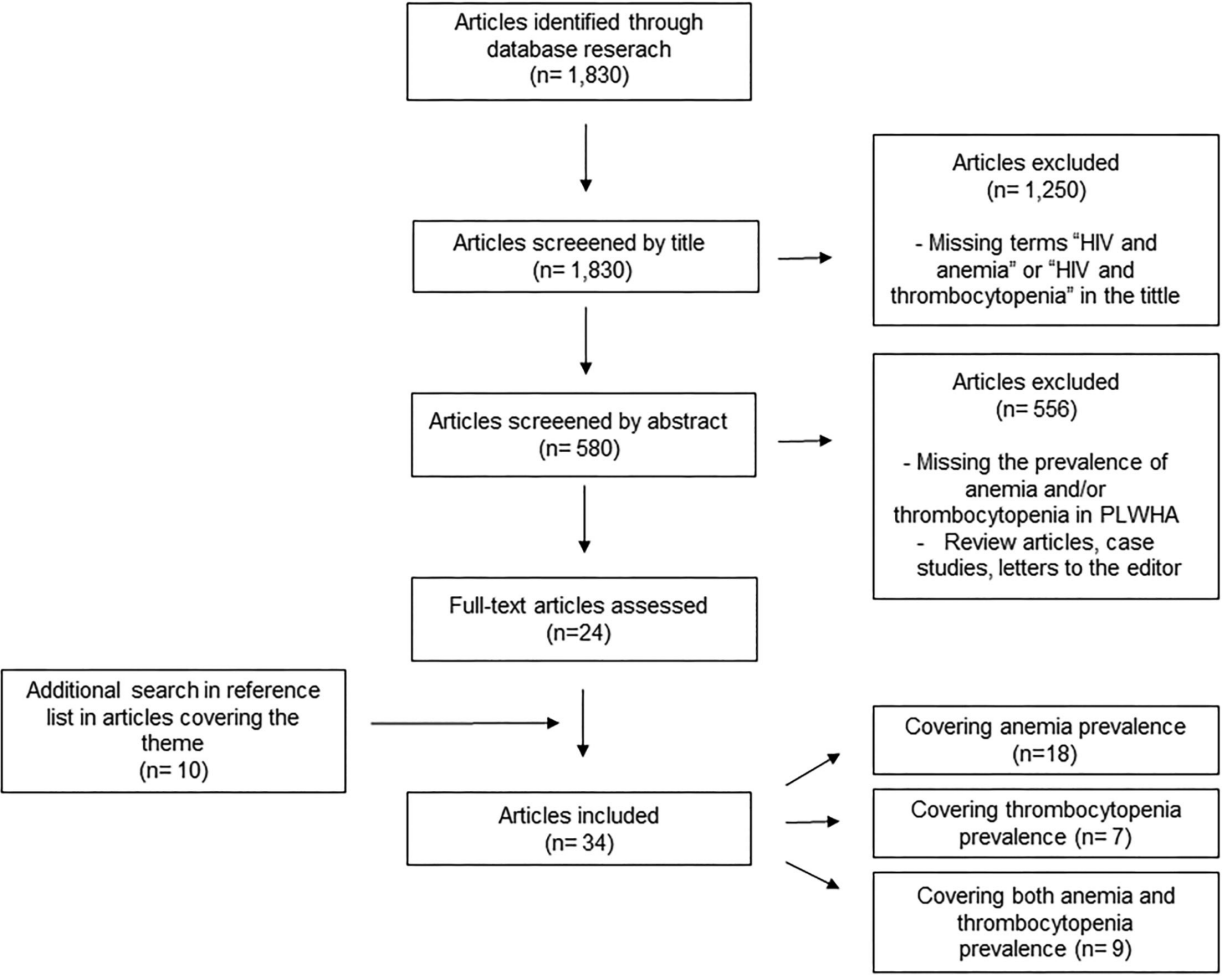
Literature selection flowchart.

First, articles that had both search terms ‘HIV *and* anemia’ and ‘HIV *and* thrombocytopenia’ in the title, published in the last 10 y, were preselected. Both authors read the abstracts of these preselected articles independently. Studies that reported the prevalence and/or hematological characteristics of anemia and/or thrombocytopenia, when these criteria were identified by both researchers individually, were finally included. Literature review articles, case studies, letters to the editor and studies that did not contemplate the scope were excluded. Finally, additional searches were made in the reference lists of articles covering the theme (Figure 1).

## Results and Discussion

We found 1830 articles from the crossing of the terms used in the search. First, we excluded 1250 articles because they were missing the terms ‘HIV and anemia’ and ‘HIV and thrombocytopenia’ in the title. Subsequently, we excluded 556 articles missing the prevalence of anemia and/or thrombocytopenia or that were either a literature review, letter to the editor or a case report, by reading the abstract. Therefore, we obtained 24 articles, to which 10 articles found in the reference lists of articles covering the topic were added. Finally, for this review we used 34 articles, 18 covering anemia prevalence, 7 covering thrombocytopenia prevalence and 9 covering both anemia and thrombocytopenia prevalence (Figure 1).

### Anemia in people living with HIV/AIDS

Our research generated 27 articles reporting the prevalence and/or hematological features of anemia in adult PLWHA in the last 10 y (Table 1): 6 cohort, 18 cross-sectional, 1 longitudinal, 1 case-control and 1 randomized clinical trial. These studies reported an overall prevalence of anemia from 6.3% to 84%^3^^,^^6^^,^^10^^,^^12^^-^^34^ in PLWHA worldwide and demonstrated they have significantly lower hemoglobin levels than healthy controls in peripheral blood.^32^

**Table 1.** Prevalence and features of anemia in people living with HIV/AIDS (PLWHA) reported in studies from different countries in the last 10 y

Study design	Sample	Main results	Place, year	Reference
Retrospective cohort study	15 126 PLWHA	- 7.2% PLWHA developed anemia during a median follow-up of 6.6 y	USA, 2020	^12^
		- 3.1% PLWHA developed severe anemia during a median follow-up of 6.6 y		
		- Older age, female gender, black race, HCV coinfection, lower CD4 cell counts, viral load ≥400 copies/mL were associated with incident anemia		
Observational cohort study	199 PLWHA with severe anemia, 45.7% on ART	- 42.2% PLWHA had very severe anemia (hemoglobin ≤50 g/L).	Malawi, 2020	^6^
		- 94% of the patients had more than two potential causes for anemia: iron deficiency (55.3%), underweight (73.9%), TB infection (41.2%), viral load >1000 copies/mL (73.9%)		
		- Overall mortality in anemic patients was 53%		
		- EBV/CMV coinfection (16.5%) associated with very severe anemia		
Retrospective cohort study	440 PLWHA on ART	- Improved mean hemoglobin level 6 mo after ART in females	Ghana, 2020	^13^
		- Non-ZDV ART-based regimen reduced odds of anemia in males		
Cross-sectional study	1456 PLWHA in 2010, 1531 PLWHA in 2012 and 1567 PLWHA in 2014	- Prevalence of anemia: 44.3%, 34.7% and 27.6% in PLWHA in 2010, 2012 and 2014, respectively	China, 2019	^14^
		- Patients with a higher level of education had a lower risk of anemia in 2014		
		- Patients receiving a longer duration of ART had a higher risk of anemia in 2014		
Longitudinal study	796 PLWHA with undetectable viral load (HIV RNA <50 copies/mL) and 2388 uninfected controls	- Prevalence of anemia significantly higher in PLWHA (6.9%) than in uninfected controls (3.4%)	Denmark, 2019	^15^
		- HIV independently associated with anemia		
Cross-sectional study	411 PLWHA, 75% on HAART	- Prevalence of anemia: 36.5%	Ethiopia, 2019	^16^
		- PLWHA >5 y, low CD4 count, infection with intestinal parasites, low BMI and being HAART-naive associated with anemia prevalence		
Cross-sectional study	320 PLWHA on ART, the regimen consisted of a combination of nucleoside and non-nucleoside reverse transcriptase inhibitors	- Prevalence of anemia: 25%	Ethiopia, 2015	^17^
		- 2.5% PLWHA had severe and 21.3% moderate anemia		
		- 45% had macrocytic anemia		
		- HAART-naive and CD4 count <200 cells/μL as independent and significant anemia predictors		
Cross-sectional study	364 PLWHA, 133 on HAART and 231 HAART-naive	- Prevalence of anemia: 51.5%	Ethiopia, 2018	^18^
		- 32.2% had macrocytic anemia		
Cross-sectional study	141 PLWHA ART-naive	- Prevalence of anemia: 6.3%	Uganda, 2018	^19^
		- CD4 count <200 cells/μL associated to anemia		
		- 89.4% had normocytic anemia		
Retrospective cohort study	197 PLWHA on ZDV-based ART and 197 non-ZDV-based ART	- Prevalence of anemia: 20.8%, 33.5% and 13% (baseline, 6 and 12 mo of ART follow-up among ZDV group)	Ethiopia, 2018	^20^
		- Prevalence of anemia: 44.2%, 18.3% and 12.4% (baseline, 6 and 12 mo of ART follow-up among non-ZDV group)		
		- 45% had macrocytic anemia in ZDV group		
		- ZDV group 3.34-fold more likely to develop severe anemia than non-ZDV group		
Cross-sectional study	54 PLWHA on ART for more than 6 wk	- Prevalence of anemia: 38.8%	Indonesia, 2018	^21^
		- 55.1% had macrocytic anemia		
		- White blood cells <5 × 10^9^ /L and CD4 <200 cells/μL were associated with higher rates of anemia		
Hospital-based cross-sectional	377 PLWHA	- Prevalence of anemia: 23%	Ethiopia, 2017	^33^
		- Being ART-naive, having treatment history with anti-TB drugs, ZDV-based ART regimen and CD4 >200 cells/μL were associated with anemia		
Cross-sectional study	307 PLWHA	- Prevalence of anemia: 59.6%	South Africa, 2017	^22^
		- CD4 count <200 cells/μL associated with anemia		
Cross-sectional study	94 PLWHA, 29 ART-naive, 31 on ZDV-based ART, 34 on non-ZDV-based ATR and 20 healthy controls	- PLWHA on ZDV-based ART had mean MCV and RDW higher than control group and ART-naive group	Nigeria, 2016	^35^
Cross-sectional cohort clinic-based study	346 PLWHA on HAART	- Prevalence of moderate to severe anemia: 40.46%	Tanzania, 2016	^23^
		- ZDV-based ART and CD4 count <200 cells/μL as strongly predictors of anemia		
		—MCV >100 fL in most anemic patients		
Comparative cross-sectional study	319 PLWHA, 219 on HAART (for at least 3 mo) and 100 HAART-naive patients	- Prevalence of anemia: 23.8%	Ghana, 2016	^24^
		- Higher prevalence of anemia in HAART-naïve patients		
		- Severe anemia related to decreased serum iron concentration		
Retrospective cohort study	1061 PLWHA on HAART	- Prevalence of anemia at baseline: 42.9%	Ethiopia, 2015	^25^
		- Prevalence of anemia significantly decreased to 20.9% at 6 mo and to 14.3% at 12 mo after HAART initiation		
Comparative cross-sectional study	234 PLWHA, 117 on HAART and 117 HAART-naive.	- Prevalence of anemia: 23.1%	Ethiopia, 2014	^26^
		- Prevalence of anemia in HAART- naive and HAART experienced PLWHA was 29.9% and 16.2%, respectively		
		- Presence of opportunistic infections, CD4 count <200 cells/μL and rural residence as predictors of anemia in HAART- naïve PLWHA		
		- HAART regimen (ZDV/3TC/NVP) and the duration of HAART as predictors of anemia for HAART-experienced groups		
Cross-sectional study	290 PLWHA, 145 on ART and 145 ART-naive	- Prevalence of anemia: 20.7%	Ethiopia, 2014	^27^
		- Prevalence of anemia was 11.7% in PLWHA on HAART; 29.7% in HAART-naive patients		
		- 58.8% of macrocytic anemia cases on PLWHA on HAART		
Cross-sectional study	500 PLWHA, 185 on HAART and 315 HAART-naive	- Prevalence of anemia in HAART-naive patients was 57.5%, significantly higher compared with PLWHA on HAART (24.3%)	Nigeria, 2013	^28^
		- The use of HAART not associated with severe anemia		
Cross-sectional study	1948 PLWHA newly diagnosed with HIV/AIDS	- Prevalence of anemia: 51.9%	China, 2013	^34^
		- Prevalence of mild, moderate and severe anemia was 32.4%, 17.0% and 2.5%		
		- Prevalence of anemia increased with age and decreasing CD4 count		
Prospective cohort study	10 259 PLWHA initiating first-line ART	- Prevalence of anemia at ART initiation: 25.8%	South Africa, 2013	^29^
		- After ART initiation, hemoglobin increase linearly related to CD4 count		
Cross-sectional study	250 PLWHA	- Prevalence of anemia: 84%	India, 2012	^10^
		- 40.4% had normocytic anemia		
		- Prevalence of anemia in PLWHA with CD4 <200 cells/μL: 91.4%		
Cross-sectional study	710 PLWHA ART-naive and 226 uninfected controls	- Higher prevalence of anemia in PLWHA (20.5%) in relation to controls (6.3%)	Rwanda, 2012	^30^
		- BMI, CD4 count 200–350 cells/μL and CD4 count <200 cells/μL were associated with anemia		
Retrospective cross-sectional	701 PLWHA	- Prevalence of anemia: 37.5%	Brazil, 2011	^31^
		- Prevalence of anemia in low CD4 count group: 61.1%		
		- Prevalence of macrocytic anemia: 45%		
		- Higher mortality rate in PLWHA with low hemoglobin concentrations		
Case-control study	48 PLWHA on ART at least 6 mo and 45 healthy subjects as controls	Lower hemoglobin levels in PLW-HIV than healthy controls	Brazil, 2010	^32^
Randomized clinical trial	1532 PLWHA ART-naive	- Prevalence of anemia: 12%	Africa, Asia, South America, Caribbean and USA, 2010	^3^
		- Anemia associated with gender (female), platelet count and country		
		- Higher prevalence of anemia in Malawi, Haiti, South Africa and Zimbabwe		

ART: antiretroviral therapy; BMI: body mass index; CMV: cytomegalovirus; EBV: Epstein–Barr virus; HAART: highly active ART; HCV: hepatitis C virus; MCV: mean corpuscular volume; NVP: nevirapine; RDW: red blood cell distribution width; 3TC: lamivudine; ZDV: zidovudine.

**Table 2.** Prevalence and features of thrombocytopenia in people living with HIV/AIDS (PLWHA) reported in studies from different countries in the last 10 y

Study Design	Sample	Main results	Place, year	Reference
Retrospective cohort study	440 PLWHA on ART	- Reduced mean platelet count 6 mo after ART	Ghana, 2020	^13^
Cross-sectional study	310 PLWHA	- Prevalence of thrombocytopenia: 19%	Cameroon, 2019	^46^
		- ART-naive PLWHA, viral load >3 log^10^ RNA/mL, CD4 count <200 cells/μL associated with higher prevalence of thrombocytopenia		
Longitudinal study	796 PLWHA with undetectable viral load (HIV RNA<50 copies/mL) and 2388 uninfected controls	- Prevalence of thrombocytopenia was significantly higher in PLWHA (5.5%) than in uninfected controls (2.7%)	Denmark, 2019	^15^
		- Thrombocytopenia independently associated with anemia		
Cross-sectional study	133 PLWHA on HAART and 231 PLWHA HAART- naive	- Prevalence of thrombocytopenia: 11.1%	Ethiopia, 2018	^18^
		- Mean platelet count higher in PLWHA with CD4 count >200 cells/μL		
Cross-sectional study	320 PLWHA on ART; regimen consisted of a combination of nucleoside and non-nucleoside reverse transcriptase inhibitors	- Prevalence of thrombocytopenia: 6.3%	Ethiopia, 2018	^17^
Cross-sectional study	141 PLWHA ART-naive	- Prevalence of thrombocytopenia: 26.2%	Uganda, 2018	^19^
		- CD4 count <200 cells/μL associated with thrombocytopenia		
Cross-sectional study	307 PLWHA	- Prevalence of thrombocytopenia: 12.1%	South Africa, 2017	^22^
Cross-sectional study	1948 PLWHA newly diagnosed	- Prevalence of thrombocytopenia: 15.6%	China, 2015	^47^
		- Lower CD4 count and HIV transmitted through blood were significantly associated with an increased risk of thrombocytopenia		
Retrospective cross-sectional study	15 030 PLWHA	- Prevalence of thrombocytopenia: 17.4%	Uganda, 2018	^45^
		- Significant association between thrombocytopenia and other cytopenias, CD4 counts, ART and deteriorating HIV stage		
		- 5% of PLWHA had antiplatelet antibodies		
Cross-sectional study	1730 PLWHA HAART-naive	- Prevalence of thrombocytopenia: 4.5%	China, 2015	^8^
		- Age ≥50 y, HCV-Ab positivity and CD4 50–199 cells/μL significantly associated with thrombocytopenia		
Cross-sectional study	290 PLWHA, 145 on ART and 145 ART-naive	- Prevalence of thrombocytopenia: 6.6%	Ethiopia, 2014	^27^
		- Increased prevalence of thrombocytopenia observed in PLWHA with CD4 count <200 cells/μL.		
Retrospective cross-sectional study	390 PLWHA HAART-naive	- Prevalence of thrombocytopenia: 5.9%	Ethiopia, 2014	^44^
		- PLWHA aged ≥50 y were 2.5-fold more likely to have thrombocytopenia		
		- PLWHA whose CD4 count <350 cells/μL were 2.6-fold more likely to have thrombocytopenia than PLWHA whose CD4 count ≥500 cells/μL		
Cross-sectional study	710 PLWHA ART-naive and 226 uninfected controls	- Higher prevalence of thrombocytopenia in PLWHA (13.5%) in relation to controls (8.6%)	Rwanda, 2012	^30^
Cross-sectional study	250 PLWHA	- Prevalence of thrombocytopenia: 18%	India, 2012	^10^
		- Increased prevalence of thrombocytopenia observed in PLWHA with CD4 count <200 cells/μL		
Retrospective cross-sectional study	5290 PLWHA	- Prevalence of thrombocytopenia: 26%	Canada, 2012	^9^
		- Prevalence of severe thrombocytopenia: 0.6%		
Retrospective cross-sectional study	55 PLWHA with thrombocytopenia	- 63.6% PLWHA with immune thrombocytopenic purpura	Brazil, 2012	^48^
		- 25.4% PLWHA with platelet production deficiency		
		- HAART improved platelets count 3 mo after starting treatment		
Randomized clinical trial	1532 PLWHA ART-naive	- Prevalence of thrombocytopenia: 7.2%	Africa, Asia, South America, Caribbean and USA, 2010	^3^
		- Higher prevalence of thrombocytopenia in India, Brazil, Malawi and USA		

ART: antiretroviral therapy; HAART: highly active antiretroviral therapy

### Anemia in people living with HIV/AIDS in different locations worldwide

Results show that different countries have different prevalence levels of anemia in PLWHA. In this context, developed countries have a lower rate of this comorbidity during HIV infection. In a recent cohort study of PLWHA receiving care at eight sites across the USA, 7.2% of individuals developed anemia after 6 y of follow-up, indicating an incidence of anemia of 1.95/100 person-years.^12^ In Denmark, a longitudinal study detected the prevalence of 6.9% of anemia; however, PLWHA evaluated had an undetectable viral load, which may have contributed to the low rate of anemia observed.^15^

On the other hand, developing Asian countries showed higher rates of anemia in PLWHA. In Indonesia,^21^ the prevalence of anemia was 38.8% whereas in India^10^ it was 84% in the cases studied. A recent Chinese study^14^ showed anemia prevalence of 44.3%, 34.7% and 27.6% in 2010, 2012 and 2014, respectively, thus showing an important decline in incidence over time. All studies conducted in Asia were cross-sectional, having a lower level of evidence than the longitudinal studies conducted in developed countries.

On the African continent, eight studies were conducted in Ethiopia, of which six were cross-sectional. In these cross-sectional studies,^16^^-^^18^^,^^26^^,^^27^^,^^33^ prevalence ranged from 20.7% to 51.5% in sample populations that contemplated PLWHA on ART and those who were ART-naïve. The two retrospective cohort studies^20^^,^^25^ showed a prevalence of anemia at baseline of ART of 20.8%, 44.2% and 42.9%, which declined after starting treatment. Surprisingly, striking differences were found in other African countries in cross-sectional studies. In Uganda,^19^ the prevalence of anemia in PLWHA who were ART-naive was only 6.3%, while in South Africa^22^ it was 59.5% and, in Nigeria, 57.5%. In PWLHA on ART, the prevalence was 40.46% in Tanzania^23^ and 24.3% in Nigeria.^35^

Corroborating these data, a randomized clinical trial of ART efficacy in Africa, Asia, South America, Caribbean and the USA^3^ showed that anemia prevalence varied significantly among these places. In this context, prevalence of anemia was highest in Malawi, Haiti, South Africa and Zimbabwe. This study provides strong evidence that the prevalence of anemia is higher in developing countries because data were collected from PLWHA with advanced untreated HIV-1 infection living in nine countries geographically distant at the time of the patient's entry into the clinical trial. In addition, to enter clinical trials, it is necessary to pass strict inclusion and exclusion criteria, which confers greater homogeneity in the PLWHA sample population in these studies than in independent cohorts. The differences in anemia rates between developed and developing countries can be explained by the differences in the levels of poverty, malnutrition and the overall poor economic state, which are accentuated mainly in African countries.^3^

### Anemia in people living with HIV/AIDS in different clinical conditions

Disease progression and ART may influence the presence of anemia during HIV infection. To explain this, one study discussed CD4^+^ T lymphocyte count as a possible predictor of anemia development in PLWHA.^26^

Different cross-sectional studies have shown that the highest prevalence of anemia occurs in PLWHA with a CD4^+^ T lymphocyte count of <200 cells/μL,^17^^,^^19^^,^^22^ characterized by peripheral immunosuppression and hematopoietic activity decline in bone marrow. A retrospective cohort study showed that a lower CD4^+^ T lymphocyte count was associated with anemia and severe anemia and that women with a CD4^+^ T lymphocyte count of <100 cells/μL were at an increased risk of developing anemia.^12^ Also, blood hemoglobin levels, mean corpuscular volume and mean corpuscular hemoglobin were shown to be lower in PLWHA with a CD4^+^ T lymphocyte count of <200 cells/μL than in PLWHA with asymptomatic HIV infection.^4^

Hemoglobin levels can also be useful in monitoring the PLWHA response to ART. A prospective cross-sectional study indicated that 0–6 mo after the start of ART, the increase in hemoglobin was significantly associated with an increase in CD4^+^ T lymphocyte count. After 6 mo of treatment, hemoglobin levels showed a sustained overall increase, regardless of the baseline CD4^+^ T lymphocyte count.^29^

In addition to CD4^+^ T lymphocyte count, viral load is a marker that predisposes to the development of anemia. The prevalence of anemia is higher in PLWHA with an HIV viral load upper than 400 or 1000 copies of HIV RNA per mL of blood.^6^^,^^12^ Thus, ART, while suppressing the individual's viral load, may indirectly improve hemoglobin levels because it improves the erythropoietic dysfunction resulting from the increased viral load. In fact, a longitudinal study^15^ revealed a prevalence of 6.9% of anemia in PLWHA with undetectable viral load, suggesting that patients with controlled disease have fewer hematological comorbidities.

The HIV treatment influences the development of anemia. Some authors have described the beneficial effect of ART on the frequency of anemia during HIV infection. A recent study conducted in a sample of 1061 individuals showed a prevalence of 42.9% of anemia before ART. However, after 6 mo of the therapeutic regimen, the prevalence of anemia was reduced to 20.9% and, after 1 y of the therapeutic regimen, this rate decreased to 14.3%.^25^ Another study in a sample of 234 PLWHA displayed an anemia prevalence of 23.1%; however, when these individuals were divided into two groups according to treatment, the prevalence of anemia in the group receiving ART was 16.2% and in the group not receiving ART it was 29.9%.^26^ In Nigeria, a study conducted with 500 PLWHA showed that the prevalence of anemia was lower and the mean hemoglobin concentration was significantly higher in patients receiving ART.^28^ In addition, there was an increase in hemoglobin levels 6 months after ART and a non-zidovudine (ZDV)-based ART regimen reduced the odds of anemia.^13^

Nevertheless, Takuva et al*.*^29^ found a prevalence of 25.8% of anemia in a population of 10 259 PLWHA in South Africa. After starting treatment in 322 of these individuals, the percentage of anemia was doubled. The therapeutic regimen used was based on ZDV, which is described as a possible inhibitor of erythroid colony-forming units, leading to decreased production of red blood cells.^10^ As a result, patients being treated with a ZDV-based ART regimen are 3.34-fold more likely to develop severe anemia than patients being treated with a non-ZDV ART regimen.^20^

Corroborating this, another study conducted a series of multivariate logistic regressions to analyze the association between the 16 most frequent combinations of ART and the presence of anemia. Results demonstrated that the treatment regimens associated with the global prevalence of anemia were abacavir/ZDV/lamivudine, ZDV/lamivudine/indinavir and ZDV/lamivudine/efavirenz. By contrast, a low prevalence of anemia was observed in subjects receiving didanosine/stavudine/efavirenz, didanosine/stavudine/nelfinavir and stavudine/lamivudine/indinavir. In summary, all ZDV-containing regimens were associated with an increased risk of anemia, with the exception of the ZDV/lamivudine/saquinavir regimen.^11^

### Hematological aspects and causes of anemia in PLWHA

In line with a narrative literature review of prevalence of anemia in PLWHA, this section will now present a comprehensive understanding of the etiology and pathophysiology of anemia in PLWHA.

Anemia in PLWHA is associated with low reticulocyte counts, demonstrating its hyporegenerative features^35^ due to hematopoietic suppression in the bone marrow. This suppression is multifactorial, reflecting the effects of viral infection, inflammation, malnutrition, malignancy and ART. In this context, HIV disrupts the bone marrow microenvironment, leading to an increase in inflammatory cytokines, immunoglobulins and acute phase proteins as a response of the host organism to viral infection. Cytokine imbalance can affect hematopoietic progenitor cells in different ways. Increased IL-6 induces augmented production of hepcidin, an important regulator of iron homeostasis. The increase of hepcidin is responsible for the retention of iron inside the macrophages and enterocytes, which leads to a decrease in serum iron concentration and, consequently, a decrease in hemoglobin production.^36^^,^^37^ In fact, severe anemia in PLWHA was related to decreased serum iron concentration.^24^ In addition, IL-1β and TNF-α cytokines inhibit the production of erythropoietin, interfering with the proliferation of erythrocyte precursor cells.^10^

Nutritional deficiencies are frequently found in PLWHA because of anorexia and gastrointestinal disorders caused by ART. Vitamin B12 deficiency is the most common nutritional deficiency and occurs in more than 30% of PLWHA as a consequence of abnormalities in vitamin B12 binding proteins.^38^ Folate deficiency occurs more frequently in advanced immunodeficiency and in the context of diarrhea and severe malabsorption states.^37^ Iron deficiency and poor distribution of iron induced by inflammation may also contribute to PLWHA anemia. A recent study showed that PLWHA with anemia had lower levels of serum iron and ferritin and that progression of HIV infection increases total iron-binding capacity.^24^

Opportunistic infections in PLWHA are an important cause of anemia development and some of these infections are traditionally related to abnormal hematopoiesis. Bacteria such as *Mycobacterium tuberculosis*, fungi like *Histoplasma* spp. and *Cryptococcus* spp., and parasites such as *Leishmania* spp. and *Plasmodium falciparum* often infiltrate the bone marrow, affecting its normal architecture and inhibiting the maturation of progenitor cells.^37^^,^^39^ In fact, hepatitis C, Epstein–Barr virus infection, cytomegalovirus infection and TB are some of the infections associated with the presence of anemia.^6^^,^^12^

Despite being predominantly a hyporegenerative anemia, hemolysis may also be responsible for anemia development in PLWHA. The formation of anti-erythrocyte antibodies as a consequence of hypergammaglobulinemia is the main cause of hemolytic anemia in PLWHA, and 18% of hospitalized PLWHA are positive for the direct Coombs test.^39^^,^^40^ Other causes of hemolytic anemia in PLWHA are microangiopathic hemolytic anemia and drug-induced anemia in patients who have glucose-6-phosphate dehydrogenase deficiency. In Africa, coinfection between HIV and malaria is the major cause of severe anemia and mortality from PLWHA.^41^

Morphologically, normocytic normochromic anemia is the most frequent^19^; however, studies also described high frequencies of macrocytosis (e.g. 55.1% and 32.2%), usually related to treatment with ZDV.^21^^,^^31^^,^^34^^,^^42^ ZDV can interfere with DNA replication and the cell division of erythroblasts, causing macrocytosis in mature erythrocytes. This phenomenon occurs because ZDV competes with natural deoxynucleoside triphosphates for binding to reverse transcriptase and deoxynucleoside triphosphates for incorporation into newly synthesized DNA viral strands, inhibiting viral reverse transcriptase and mammalian cellular DNA polymerase. The development of macrocytosis in PLWHA may signify adherence to ZDV antiretroviral regimens.^43^

Anemia is one of the most common abnormalities in PLWHA and is associated with a low quality of life and high death rates.^6^ Despite the presence of comorbidities aggravating anemia, HIV infection was independently associated with the anemic condition.^15^ Although treatment improves hemoglobin levels, even patients on ART have a significant prevalence of anemia. It is essential to ensure that a blood count is regularly performed on all patients, in order to monitor the progression of anemia and to plan interventions to reduce the potential risks associated with this comorbidity. In addition, the choice of ART is essential for the prevention of anemia development, considering that ZDV-based regimens have shown a strong association with the anemic condition. Since low hemoglobin is an established adverse prognostic marker, prompt identification of anemia may result in improved morbidity and mortality of patients initiating ART.

### Thrombocytopenia in people living with HIV/AIDS

Thrombocytopenia is considered the first hematological manifestation of HIV infection. The presence of thrombocytopenia may change according to the CD4^+^ T lymphocyte count, the age, the presence of HCV/HBV coinfection, the presence of opportunistic infections and the ART treatment. Occurrence of thrombocytopenia is a predictor of morbidity and mortality in PLWHA, leading to an accelerated progression to AIDS.^37^^,^^44^^,^^45^ Our research generated 17 articles reporting the prevalence or characteristics of thrombocytopenia in adult PLWHA in the last 10 y (Table 2): 1 cohort, 1 longitudinal, 1 randomized clinical trial and 14 cross-sectional studies. These studies reported an overall prevalence of thrombocytopenia from 4.5% to 26.2%.^3^^,^^8^^-^^10^^,^^13^^,^^15^^,^^17^^-^^19^^,^^22^^,^^27^^,^^30^^,^^44^^-^^48^

### Thrombocytopenia in people living with HIV/AIDS in different locations worldwide

Our research shows that different countries have different prevalence levels of thrombocytopenia in PLWHA. Different to the findings above with regard to anemia, the prevalence of thrombocytopenia does not appear to be higher in developing countries than in developed countries.

An earlier cross-sectional study in Canada showed a prevalence of 26% of thrombocytopenia.^9^ A longitudinal study conducted in Denmark detected a prevalence of 5.5% of thrombocytopenia. However, PLWHA evaluated had an undetectable viral load, which may have contributed to the low rate of thrombocytopenia observed.^15^

On the African continent, cross-sectional studies with small sample sizes conducted with PLWHA on ART and those who were ART-naïve demonstrated 11.1% and 6.6%, respectively, of thrombocytopenia in Ethiopia. However, a cross-sectional study with a large sample size conducted with PLWHA on ART and those who were ART-naïve demonstrated 17.4% of thrombocytopenia in Uganda. Besides, cross-sectional studies conducted with only PLWHA who were ART-naïve demonstrated 26.2% of thrombocytopenia in Uganda, 5.9% in Ethiopia^44^ and 13.5% in Rwanda.^30^

In Asia, a cross-sectional study conducted in India showed a prevalence of 18% of thrombocytopenia. A cross-sectional study developed in China, with a large sample of newly diagnosed PLWHA, revealed a prevalence of 15.6% of thrombocytopenia,^47^ while another study conducted in the same country with a large sample of PLWHA who were ART-naïve found a prevalence of only 4.5% of thrombocytopenia.^8^

Corroborating these data, a randomized clinical trial of ART efficacy in Africa, Asia, South America, the Caribbean and the USA^3^ showed an overall prevalence of thrombocytopenia of 7.2%, with the highest levels of prevalence in India, Brazil, Malawi and the USA.

### Thrombocytopenia in people living with HIV/AIDS in different clinical conditions

An individual's degree of immunosuppression and use of ART can influence thrombocytopenia development. In this context, a study carried out in Uganda with 400 individuals reported a prevalence of 15.3% of thrombocytopenia in PLWHA with a CD4^+^ T lymphocyte count of <50 cells/μL, 6.6% in PLWHA with a CD4^+^ T lymphocyte count of 50–200 cells/μL, 9.1% in PLWHA with a CD4^+^ T lymphocyte count of 200–350 cells/μL and 0% in PLWHA with a CD4^+^ T lymphocyte count of >350 cells/μL.^49^ Corroborating these results, a study carried out in Ethiopia found similar results, with a higher prevalence of thrombocytopenia in PLWHA with a CD4^+^ T lymphocyte count of <200 cells/μL.^27^ By contrast, a study of 710 Rwandan women living with HIV/AIDS found no significant difference between the frequencies of thrombocytopenia when the groups were stratified according to CD4^+^ T lymphocyte count, suggesting a non-association of CD4^+^ T lymphocytes and thrombocytopenia in this population.^30^

Moreover, the age of individuals may influence the development of thrombocytopenia during HIV infection. There is an increase in the prevalence of thrombocytopenia according to advancing age, which can be explained by the higher incidence of myelodysplasia in older patients.^8^^,^^37^

Prior to the implementation of ART, there was a higher prevalence of thrombocytopenia in PLWHA.^50^ This leads to the conclusion that adherence to the therapeutic regimen influences the development of thrombocytopenia. A recent study shows that, among those PLWHA who were ART-naïve, the prevalence of thrombocytopenia is 64.6%, whereas among PLWHA on ART, the prevalence of thrombocytopenia is 6.9%.^46^ A study carried out in Uganda showed that PLWHA on ART had a prevalence of 13% of thrombocytopenia, while in the absence of ART the prevalence was 17.8%. What is more, this study indicated the positive effect of ZDV on platelet homeostasis, while co-trimoxazil, used to treat opportunistic bacterial infections, can be considered a predictor for accelerated thrombocytopenia.^45^ Corroborating these results, a study conducted with 400 PLWHA showed ZDV as the best treatment for HIV-related thrombocytopenia.^49^ The positive effect of ART in improving platelet count shows that there is a relationship between the rate of viral replication and the induction of thrombocytopenia.

### Hematological aspects and causes of thrombocytopenia in PLWHA

In line with a narrative literature review of the prevalence of thrombocytopenia in PLWHA, this section will now present a comprehensive understanding of the etiology and pathophysiology of thrombocytopenia in PLWHA.

The causes of platelet changes in PLWHA are multifactorial and may occur as a result of peripheral platelet destruction or decreased platelet production.^37^ A study carried out in Brazil with 55 PLWHA with thrombocytopenia revealed that 63.6% of patients had idiopathic thrombocytopenic purpura while 25.5% had a platelet production deficiency, both secondary to HIV infection.^48^

Peripheral destruction usually occurs at the onset of infection due to cross-reactivity between glycoprotein 120 in the virus envelope and platelet glycoprotein IIIa. This antibody cross-reactivity promotes platelet capture and lysis in the reticuloendothelial system of the spleen or early apoptosis, resulting in idiopathic thrombocytopenic purpura or immune thrombocytopenic purpura.^51^ A study conducted in Canada showed that 0.6% of PLWHA developed severe immune thrombocytopenic purpura, of whom 55% presented clinical bleeding and 33.3% were ART-naïve.^9^ Similarly, 5% of PLWHA had antiplatelet antibodies in a large study developed in Uganda.^45^

By contrast, the decrease in platelet production usually occurs at more advanced stages of AIDS. Megakaryocytes in bone marrow express CD4 receptors and co-receptors essential for HIV infection and studies demonstrate that megakaryocytes internalize HIV and express viral RNA.^50^ As a result of the HIV infection, immature megakaryocytes decline the thrombopoietin c-Mpl receptor expression.^51^ Thereby, megakaryocytic maturation is disrupted due to impaired signaling of colony-forming units of megakaryocytes growth.^52^ HIV-infected megakaryocytes also exhibit morphological changes and an increased rate of apoptosis.^53^

Coinfection with hepatitis C (HCV) and B (HBV) viruses can also induce thrombocytopenia in PLWHA due to the possible presence of liver damage, responsible for reducing thrombopoietin production. A study carried out with 38 PLWHA reported that patients with a normal platelet count had serum thrombopoietin levels higher than in those who had severe thrombocytopenia. In addition, all patients with severe thrombocytopenia tested positive for antibodies against the hepatitis B virus core antigen.^54^ Furthermore, a recent study showed that the prevalence of thrombocytopenia was 10.2% in PLWHA who had HCV coinfection, higher than those found in PLWHA patients who did not have hepatitis C (3.9%).^8^

Considering that thrombocytopenia is the first hematological manifestation of HIV infection and that it has a strong relationship with the development of AIDS, platelet count can predict the evolution from asymptomatic HIV infection to AIDS. Monitoring thrombocytopenia development is even more essential in PLWHA with HCV and HBV coinfections because they may aggravate thrombocytopenia in these patients. ART coverage appears as an independent factor in preventing the occurrence of thrombocytopenia, justifying universal treatment for all PLWHA.

### Conclusion

**Figure 2.**
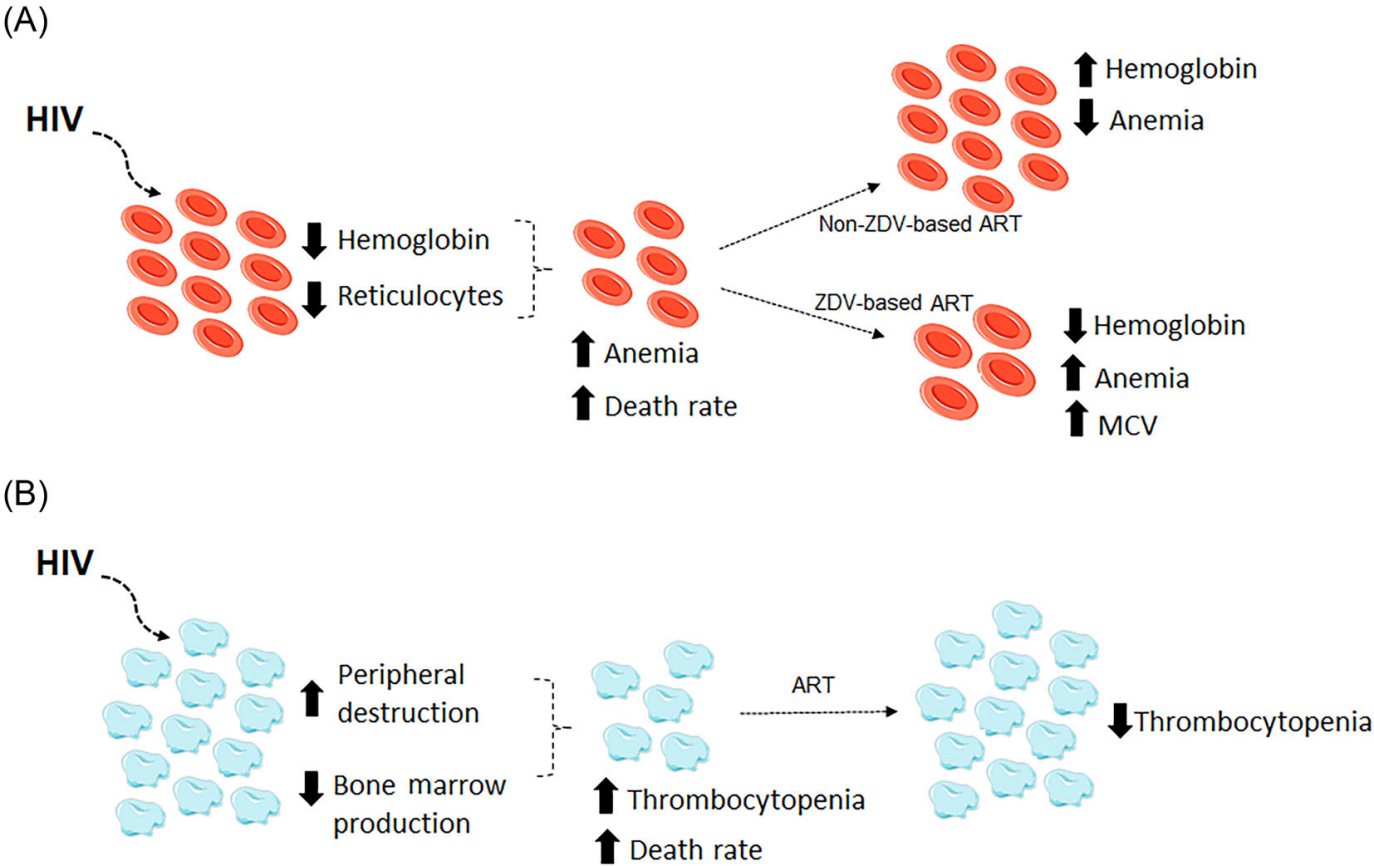
Main effects of HIV infection on erythrocytes and platelets. (A) HIV infection leads to an increase in the prevalence of hyporegenerative anemia, which is evidenced by a decrease in both reticulocyte count and hemoglobin levels. Non-based antiretroviral therapy (ART) regimens restore hemoglobin levels and decrease anemia rates; however, zidovudine (ZDV)-based ART regimens induce anemia and macrocytosis. (B) HIV infection leads to an increase in the prevalence of thrombocytopenia, which may be the result of both the immune destruction of platelets in the periphery and the decrease in their production in bone marrow. ART regimens restore platelet count and decrease thrombocytopenia rates. MCV, mean corpuscular volume.

HIV infection can induce several hematological manifestations, mainly anemia and thrombocytopenia (Figure 2). Because anemia and thrombocytopenia are treatable comorbidities associated with increased mortality among PLWHA, physicians should be aware of these risk factors. Hemoglobin levels and platelet count should be monitored routinely, especially among PLWHA with a CD4^+^ T lymphocyte count of <200 cells/μL, because AIDS development is strongly associated with these hematological manifestations. These data have an important implication because they indicate the need to start treatment to reduce the burden of anemia and thrombocytopenia in PLWHA, resulting in reduced morbidity and mortality of patients.
